# Can Big Data and Machine Learning Improve Our Understanding of Acute Respiratory Distress Syndrome?

**DOI:** 10.7759/cureus.13529

**Published:** 2021-02-24

**Authors:** Sanket Bhattarai, Ashish Gupta, Eiman Ali, Moeez Ali, Mohamed Riad, Prakash Adhikari, Jihan A Mostafa

**Affiliations:** 1 Internal Medicine, California Institute of Behavioral Neurosciences & Psychology, Fairfield, USA; 2 Research, California Institute of Behavioral Neurosciences & Psychology, Fairfield, USA; 3 Internal Medicine, Piedmont Athens Regional Medical Center, Athens, USA; 4 Psychiatry, California Institute of Behavioral Neurosciences & Psychology, Fairfield, USA

**Keywords:** big data, machine learning, acute respiratory distress syndrome, artificial intelligence in medicine, ards, analysis of big data, disease prediction

## Abstract

Acute respiratory distress syndrome (ARDS) accounts for 10% of all diagnoses in the Intensive Care Unit, and about 40% of the patients succumb to the disease. Clinical methods alone can result in the under-recognition of this heterogeneous syndrome. The purpose of this study is to evaluate the role that big data and machine learning (ML) have played in understanding the heterogeneity of the disease and the development of various prediction algorithms. Most of the work in the field of ML in ARDS has been in the development of prediction models that have comparable efficacies to that of traditional models. Prediction algorithms have been useful in identifying new variables that may be important to consider in the future, supplementing the unknown information with the help of available noninvasive parameters, as well as predicting mortality. Phenotype identification using an unsupervised ML algorithm has been pivotal in classifying the heterogeneous population into more homogenous classes. Big data generated from ventilators in the form of ventilator waveform analysis and images in the form of radiomics have also been leveraged for the identification of the syndrome and can be incorporated into a clinical decision support system. Although the results are promising, lack of generalizability, “black box” nature of algorithms and concerns about “alarm fatigue” should be addressed for more mainstream adoption of these models.

## Introduction and background

Acute respiratory distress syndrome (ARDS) occurs in 10% of all Intensive Care Unit (ICU) patients and, unfortunately, 40% of the patients with ARDS die [1]. ARDS has been defined according to Berlin’s definition based on timing, chest imaging findings, the origin of edema, and oxygenation status [2]. This diagnosis, similar to other diagnoses made in the present-day medical practice, is heavily dependent on clinical expertise, medical imaging, and lab values of biofluids [3].

When clinical methods alone are employed for the detection of ARDS, it can be missed or delayed in a significant proportion of patients [4]. Some patients with ARDS have a better prognosis than others: this phenomenon can be partly explained by the differences in the subphenotype within the ARDS cohorts along with other demographic factors such as age, race, and the difference in socioeconomic status and ventilatory management factors [1]. Because prompt diagnosis and prevention play a decisive role in the treatment outcomes of patients presenting with ARDS, clinical methods alone may not be sufficient in recognizing this increasingly heterogeneous condition [1,5].

As we enter the age of digitalization, vast amounts of data are being created as a by-product of digital devices at no additional cost. For example, the ventilators used in the ICU produce data, including pressure-time, flow-time, and volume-time data, at no additional cost. When accumulated over a prolonged period, this data becomes huge and is aptly called a type of big data. Although the innovations related to data analytics have remained relatively limited in the clinical discipline, interest in big data and its potential application in clinical care has substantially increased [6]. However, there is a gap between interest and utilization. The data generated remain underutilized due to both new and experienced clinicians’ inability to manage the enormous amounts of data [7]. Data science being the field of study devoted to the derivation of knowledge from complex data is distinctly applicable in the critical care setting [8].

Traditional statistical interpretation of data has been around for centuries and forms the backbone of current scientific medical researches. Although both machine learning (ML) and statistics can be used for inference and prediction, ML is more focused on prediction and statistics has a long-focused preference for inference [9]. ML refers to the field of study that centers around using computers for learning from available data and the development of algorithms that make this learning a reality [8]. ML models can, however, give extreme results. On one hand, ML models can outperform traditional statistical models by discovering the complex non-linear relationship between patient and disease state, while, on the other hand, it can result in erratic predictions if used in situations beyond the stretch of training data [10].

In this article, we aim to explore the role of big data and ML in advancing our understanding of ARDS. We also aim to investigate the opportunities that they may provide regarding triage of suspected patients, early diagnosis, prediction of complications, and obstacles that lie ahead regarding the usage in the everyday clinical scenario.

## Review

Big data and machine learning: A brief introduction

Big data refers to large datasets that are generated at high volume, velocity, or variety that are too large for the traditional data-processing systems and therefore require new technologies [11,12]. One example of such large data is Medical Information Mart for Intensive Care III (MIMIC III), which contains de-identified information from over 40,000 patients from the critical care unit of Beth Israel Deaconess Medical center from 2001 to 2012 [13]. Another example would be ventilators that generate a large amount of pressure-time and flow-time data per unit time. When data are collected even over a short period, this also becomes an example of big data.

**Figure 1:**
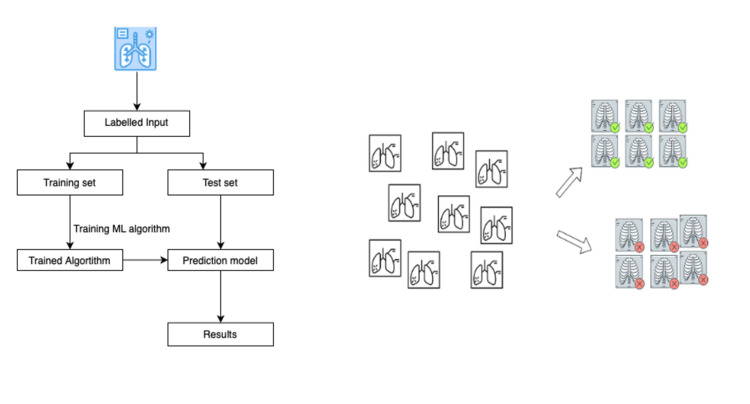
The first flowchart depicts the process of supervised ML algorithm used to create a prediction model. The second flowchart depicts the use of unsupervised ML algorithm to create clusters based on the inherent characteristics. ML, machine learning

ML can be broadly divided into supervised and unsupervised learning. Supervised ML methods are used when there are labeled input data and the output is known [14]. Suppose we need to create a prediction model for predicting the mortality of the patients based on various parameters. In this case, the output is “mortality” and the observations are labeled in the form of various parameters. This is a perfect use case scenario for supervised ML models. On the other hand, unsupervised ML methods are used when there are unlabeled observations and the output is unknown. For example, we supply the data to the machine and it automatically groups the data into certain clusters based on the inherent characteristics. Figure 1 depicts a simplified process of how supervised and unsupervised learning work.

Applications of big data and machine learning in acute respiratory distress syndrome

Prediction Models

The most widespread use of big data and ML algorithms in critical care has been in the development of prediction models [8]. Models for predicting ARDS have been created either using the Electronic Health Record (EHR) from the hospitals [15], available data from datasets like MIMIC III [13,16] and ARDS network trials [17]. Rehm et al. developed a relatively cheap method to acquire large amounts of ventilator waveform data (vWD) using a low-cost microcomputer, Raspberry Pi, attached to the ventilator [18].

Algorithms used for the prediction of ARDS include mostly supervised ML algorithms including regression [15], classification [17], decision trees [17,19], or neural network [20]. There have also been attempts to identify ARDS with the help of unstructured data such as text from radiology reports using natural language processing and ML [21]. Some models also utilize a combination of different methods known as ensemble methods [5,19]. Ensemble methods are particularly useful in decreasing overfitting: where the model performs well in the training data but the result is not replicated in the test data.

Identifying variables: ML algorithms have been used not only to identify whether a person will develop ARDS but also to identify what variables might be important to consider in patients prone to developing ARDS. For example, Ding et al. used the random forest approach to recognize the best set of predictors out of 42 different variables measured on day one of admission in 296 patients admitted to the ICU [19]. The model highlighted the importance of four new biomarkers: decreased minimum hematocrit, glucose, sodium, and increased white blood cell count. This gives a better insight into which other variables may be considered in the future for the development of a prediction scoring system.

Predicting Pao_2_/Fio_2_ (P/F) ratio using noninvasive physiological parameters: Berlin definition requires a P/F ratio for the diagnosis and classification of ARDS. The calculation of the P/F ratio has its own set of difficulties. One problem is that regular monitoring of blood gas requires indwelling catheters which are difficult to care for daily and are not easy to operate in some distinct patients, such as newborns and elderly patients [22]. In addition, conventional arterial blood gas (ABG) analysis cannot keep track of the development of ARDS in patients in real time. This creates difficulty for clinicians to diagnose the patients early and adopt suitable respiratory therapy strategies [23].

The model developed by Yang et al. (2020) showed that it is possible to predict the P/F ratio using noninvasive physiological parameters [16]. This model has been demonstrated to be effective in categorizing the oxygenation status P/F (≤300 or >300) using the parameters obtained from ventilators and physical examination of patients without blood gas analysis. This type of model serves as a footprint for the development of models that can be deployed in predicting the P/F ratio in real time without the need for regular ABG analysis. Furthermore, the model also can be used in rural areas of low-middle-income countries, where the equipment for ABG analysis may not be available.

Prediction of mortality: Prognostic evaluation of admitted patients is another area where ML algorithms have been developed. There are existing prognostic models for predicting mortality in the ICU, the one used relatively commonly includes Acute Physiology and Chronic Health Evaluation (APACHE) III [24] scoring, which is relatively cumbersome to calculate [17]. Classification tree algorithm developed by Brown et al. uses a simple rule to differentiate acute lung injury (ALI) patients according to the risk of hospital mortality using four variables: age, minute ventilation or respiratory rate, blood urea nitrogen, and shock [17]. Furthermore, it is claimed to be relatively easy to use and does not require mathematical operations. Another approach using a genetic algorithm was developed by Zhan, who identified seven important variables (age, AIDS, leukemia, metastatic tumor, hepatic failure, lowest albumin, and FiO_2_) for the prediction of mortality in ARDS patients [20]. These approaches achieved comparable performance to widely used APACHE III scoring in predicting mortality of patients in hospitals admitted with ARDS. Table 1 summarizes various studies describing the prediction models in ARDS.

**Table 1:** Summary of the models created for prediction. AUROC of only the best performing algorithm in each study have been included. The two values for studies in the size of dataset column indicate training set and test set where applicable. AUROC: area under receiver operating curve; ARDS: acute respiratory distress syndrome; APACHE III: Acute Physiological and Chronic Health Evaluation III; NLP: natural language processing; ML, machine learning; ICU: intensive care unit; EHR: Electronic Health Record; GA: genetic algorithm; MIMIC III : Medical Information Mart for Intensive Care III; P/F: PaO_2_/FiO_2_

Author(s)	Dataset used	Size of dataset	AUROC	Conclusion of study
Brown et al. (2011) [17]	ARDS network trial	(1,800, 222)	0.71 vs. 0.73 (APACHE III)	Simple classification rule was developed that stratified patients according to hospital mortality which was comparable to widely used APACHE III
Afshar et al. (2018) [21]	Data from the 533 patients admitted to certain wards of a tertiary medical center	533	0.8	NLP and ML were used to build a computable phenotype of ARDS
Christie et al. (2019) [5]	Observational cohort data	1,494	0.84-0.89	Superlearner fits provide versatile means of helping clinicians integrate big data on severely injured patients into real-time, dynamic decision-making support
Ding et al. (2019) [19]	ICU data of patients admitted to five different centers in Beijing	296	0.82	A model for predicting ARDS was developed in Chinese patients which included 11 features
Zeiberg et al. (2019) [15]	Single-center EHR data	(1,621, 1,122)	0.81	Feasibility of ML models to risk stratify ARDS patients solely based on EHR data was demonstrated
Zhang (2019) [20]	Secondary analysis of two randomized controlled trials conducted across 44 hospitals	1,071	0.821 vs. 0.665 (APACHE III)	A model based on neural network using GA was developed which outperformed the conventional scoring system for predicting mortality in ARDS patients
Yang et al. (2020) [16]	MIMIC III	(6,601, 2,101)	0.9128	An algorithm based on patients’ noninvasive physiological parameters to estimated P/F ratio was developed

Phenotyping

The unsupervised machine learning algorithm can help to identify clusters of the population that have similar inherent characteristics. Due to some intrinsic difference, some patients are more likely to develop complications and may differ in response to treatment. One of the landmark studies using latent class analysis, which is an unsupervised ML algorithm, was performed by Calfee et al., who identified two distinct subphenotypes of ARDS: hyperinflammatory and hypoinflammatory. These two phenotypes differed in the severity of inflammation, presence of shock, and metabolic acidosis [25]. They were also found to have different responses to treatment in positive end-expiratory pressure strategies. The two phenotypes were further found to respond differently to a randomly assigned fluid strategy [26].

Cluster-based methods incorporate various analytical techniques that mainly focus on identifying clusters of observations with similar characteristics [27]. Bos et al. found that it is possible to cluster the patient population into two biological phenotypes: “uninflamed” and “reactive,” based solely on four biomarkers (interleukin-6, interferon gamma, angiopoetin 1/2, and plasminogen activator inhibitor-1) with the help of cluster analysis [28]. These studies have been deemed pivotal, especially in identifying groups of the population that can be enrolled in various randomized control trials. As the research continues to grow in the field of ARDS, new biomarkers have emerged to be informative in the past several years. New algorithms developed in the future may identify these biomarkers to result in more comprehensive subphenotypes for classification of ARDS [25].

Waveform Analysis

Patients with ARDS often need ventilatory support with lung-protective mechanical ventilation strategies [29]. Patient-ventilator asynchrony can lead to worsening of the ventilation in already compromised lungs. The flow, volume, and airway pressure data collected from the ventilators may help to grossly estimate the respiratory system mechanics and track the effects of disease progression and various therapeutic interventions [30].

Rehm et al. developed a relatively cheap method of collecting a large amount of vWD, which was then utilized in different ML models to identify patients likely to develop ARDS using physiological signatures and patient-ventilator dyssynchrony during ventilation [18]. One of the random forest classifier models used was found to have superior performance than that reported by ICU physicians, with a specificity of 92% and area under receiver operating curve (AUROC) of 0.88. Sottile et al. also developed a model using various ML algorithms to detect ventilator dyssynchrony in patients with or at risk of ARDS [31].

Adams et al. developed an open-source method to acquire vWD and then created a multi algorithmic platform (ventMAP) for automatic recognition of off-target ventilation (OTV) in critically ill patients [32]. The model was shown to accurately identify harmful forms of OTV, and that artifact correction was achieved with the improvement of specificity of clinical event detection without a tradeoff of sensitivity. These works are proof of concepts for the development of a clinical decision support system (CDSS) for detecting ventilator dyssynchrony, which can be used to notify clinicians in real time when it occurs.

Image Analysis and Radiomics

Image analysis is a critical component of the diagnosis of ARDS. An ML-based approach developed by Solti et al. was found to be comparable to physician-annotated chest X-ray reports for the classification of ALI [33]. Radiomics refers to the process of conversion of digital medical images to usable high-dimensional data, which is inspired by the concept that biomedical images contain information that depicts underlying pathophysiology and that these relationships can be disclosed through quantitative image analyses [34]. Chen et al. constructed a noninvasive ARDS existence monitoring model using quantitative and radiomics analysis of chest computed tomography images for coronavirus disease 2019 patients [35]. The radiomics model used a multistep process with least absolute shrinkage and selection operator regression used to obtain the optimized subset of features to construct the final radiomics model [35].

Other “Omics” Data and Precision Medicine

“Omics” data refer to the data obtained from modern molecular techniques, including genomics, transcriptomics, proteomics, metabolomics, and microbiomics [8]. These types of data in conjunction with the big data analytics method have given rise to precision medicine, which has been proclaimed to be unprecedented in the field of biomedicine [36]. Single nucleotide polymorphisms have been identified with the help of targeted sequencing of candidate genes. Such polymorphisms are associated with either favorable or unfavorable outcomes in ARDS [37]. An example of genomic analysis is gene expression analysis done by Dolinay et al. in critically ill patients that yielded useful information about the strong correlation of IL-18 with ARDS risk as well as indices of morbidity and mortality [38]. When the data related to genomic analysis become more widespread and easily available, such analyses may become more commonplace. This may help in finding out other useful clinical biomarkers that can be readily used at the bedside.

Challenges, Limitations, and Way Forward

Clinical implementation of data-driven system requires the knowledge that the models that have been developed and have significant impact in the population for which it is intended to be used [8]. As the datasets used to create prediction models of ARDS were mostly from a single center [15,16], used stringent exclusion criteria [17], and were from a certain ethnicity or age of patients [19,16], there is a concern that the available models may not perform well in other institutions. Hence, due to the possibility of a lack of generalizability, they need to be tested in the future. Furthermore, the method of documentation may not be uniform across various institutions. This may lead to differences in EHR- based models [15]. The advancement in the field of ML means that there will be increasing numbers of new algorithms developed in the future. The inclusion of new algorithms in the models may improve prediction as well [5].

Although it may be tempting to look at the AUROC and p-values of the data-driven system and call it effective, clinicians and researchers assessing such a system must be aware that the measure of effectiveness goes beyond such measures of performance alone [8]. The prediction models described in the context of ARDS have achieved comparable efficacies to traditional models. They have also been claimed to be easy to use. However, most of the models are proof of concept and need to undergo refinement before integrating them into real clinical practice.

While training a model, it may be difficult to label each case of ARDS accurately as it is a complex clinical diagnosis. This may introduce label uncertainty in the models. There have been several attempts to reduce this type of label uncertainty. Reamaroon et al. created a method tested on real-world data that implemented with support vector machines (SVM) to account for such type of label uncertainty, which was shown to provide a meaningful improvement in the algorithm compared to the traditional SVM algorithms [39].

The general limitations of the use of ML in the field of medicine apply to the prediction models developed for ARDS as well. ML models may have greater explanatory power than the linear statistical methods, but when the models are used in situations that extrapolate beyond the scope of training data, they can give rise to “black box” models that do not support clinical comprehension [10]. Careful choice of appropriate ML algorithms and diligent and meticulous evaluation of models may help curb the problems [10]. Clinicians also fear the possibility of “alarm fatigue” which creates an unsafe patient environment because a life-threatening event may be missed due to sensory overload created by alerts, especially in the ICU setting [40]. ML models created for integration into the CDSS should strive towards decreasing this problem and not adding to it. There are concerns regarding the intrinsic inequities in the available data in healthcare. As the ML models are built upon the existing data, such inequities are expected to be multiplied. Hence, the process of algorithm development should include physicians and data scientists from diverse background so that such inequities can be addressed appropriately [41].

## Conclusions

ARDS is a heterogeneous syndrome with a high mortality rate. Acquisition of large amounts of data at relatively low cost has opened up the possibility of exploration of big data in deciphering this complex syndrome. Big data and ML have been used in identifying subphenotypes of ARDS which are different in terms of clinical presentation and treatment responses. Prediction of the disease occurrence and mortality have been done using various algorithms that have comparable efficacies to the existing traditional models. Ventilator waveform analysis has the prospect of use in developing various CDSSs for real-time notification to the treating physicians. Image analysis using ML approaches can be used in resource-limited settings where the human resource for the evaluation of such images is unavailable. Although the progress has been promising, there are impediments to the integration into real clinical practice. The “black box” nature of the algorithms is likely to be met with skepticism from the clinicians. A further test of these algorithms in various settings may probably overcome the fear of lack of generalizability. Furthermore, “alarm fatigue” and “algorithmic bias” should be sufficiently addressed for wider acceptance of these models. However, as these problems are solved, we can envision a future where patients with ARDS have a different outcome when man and machine are working in tandem.
